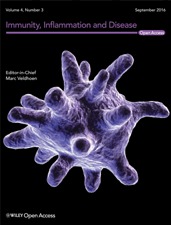# Issue Information

**DOI:** 10.1002/iid3.83

**Published:** 2016-08-30

## Abstract